# Plasma membrane aquaporins of the PIP1 and PIP2 subfamilies facilitate hydrogen peroxide diffusion into plant roots

**DOI:** 10.1186/s12870-022-03962-6

**Published:** 2022-12-05

**Authors:** David Israel, Seong Hee Lee, Thomas Matthew Robson, Janusz Jerzy Zwiazek

**Affiliations:** 1 Organismal and Evolutionary Biology (OEB), Viikki Plant Science Centre (ViPS), University of Helsinki, PO Box 65, 00014 Helsinki, Finland; 2 Department of Renewable Resources, University of Alberta, 438 Earth Sciences Building, 11223 Saskatchewan Drive NW, Edmonton, AB T6G 2E3 Canada; 3 The National School of Forestry, University of Cumbria, Rydal Road, Ambleside, LA22 9BB UK

**Keywords:** Aquaporin, PIP, Hydrogen peroxide, Root length, Arabidopsis thaliana, Oxidative stress

## Abstract

**Background:**

The permeability of plasma membrane aquaporins (PIPs) to small solutes other than water greatly diversifies their potential functions in plant development and metabolic processes. One such process is stress signalling in which hydrogen peroxide (H_2_O_2_) plays a major role. Based on transport assays carried out in yeast, there are differences in the degree to which PIPs of *Arabidopsis thaliana*, are permeable to H_2_O_2_ and thus they may differentially facilitate transmembrane diffusion. Here, we test whether specific PIPs aid in the transmembrane diffusion of H_2_O_2_ to such an extent that knocking-out PIPs affects plant phenotype. We examined changes in growth and morphology, including biomass accumulation, root system architecture and relative water content, as well as gas exchange, across two H_2_O_2_ treatments in knockout mutants of *A. thaliana*.

**Results:**

We could infer that PIP-type aquaporins are permeable to H_2_O_2_ *in planta* and that this permeability is physiologically relevant in a plant’s response to oxidative stress. In particular, the lack of functional PIP2;3 confers resistance to exogenously applied H_2_O_2_ indicating that it facilitates H_2_O_2_ entry into root cells. Additionally, PIP1;1 and PIP2;6 were found to facilitate H_2_O_2_ diffusion, while PIP2;2 is required for proper root growth under controlled conditions.

**Main findings:**

We conclude that PIPs are physiologically relevant conduits for H_2_O_2_ diffusion in the *A. thaliana* roots and participate in the regulation of stress responses.

**Supplementary Information:**

The online version contains supplementary material available at 10.1186/s12870-022-03962-6.

## Background

In addition to facilitating water movement across membranes, plasma membrane intrinsic proteins (PIPs) are permeable to other small molecules, including hydrogen peroxide (H_2_O_2_) [1–3]. This potentially endows them with a role in stress responses and signalling through the plant. This is because H_2_O_2_ mediates a variety of metabolic processes, such as apoptosis and pathogen defence when produced in response to a stressor or stimulus [4–7]. To initiate stress responses, H_2_O_2_ must diffuse across cellular membranes. It is electrochemically very similar to the water molecule [8] and thus likely to use the same diffusion pathways. Previous studies have indeed demonstrated that PIPs of *Arabidopsis thaliana* are permeable to H_2_O_2_ when expressed in yeast cells (Table 1) [1–3, 7, 9]. In fact, to date, aquaporins are the only known H_2_O_2_ transporters across phospholipid membranes [10].

**Table 1:** Plasma membrane aquaporins examined in this study and their H_2_O_2_ permeability in yeast. The effect of exogenous H_2_O_2_ application to the roots on the gene expression of the individual PIPs was reported by Hooijmaijers et al. [1] for a concentration of 1 mM in a hydroponic solution. ^1^ Dynowski et al. (2008) [2], ^2^ Hooijmaijers et al. (2012) [1], ^3^ Wang et al. (2020) [11], ^4^ Groszmann et al. (2021) [3]

Aquaporin	H_2_O_2_ permeability	Effect of exogenous H_2_O_2_
*At*PIP1;1	None^1,2,3^/High^4^	No effect
*At*PIP1;2	None^2,3^/High^4^	No effect
*At*PIP1;3	None^2,3^/High^4^	No effect
*At*PIP2;2	High^2^/Medium^3,4^	Downregulated in roots
*At*PIP2;3	Low^2^/Medium^3,4^	Downregulated in roots
*At*PIP2;4	High^1,2,3^/Medium^4^	Downregulated in roots
*At*PIP2;5	High^2^/Medium^3,4^	Downregulated in roots
*At*PIP2;6	Low^2,4^/Medium^3^	No effect

Aquaporins are channel proteins with six membrane-spanning units connected by three loops (A, C and E) on the apoplastic side and two loops (B and D) on the cytoplasmic side. Loops B and E contain a highly conserved asparagine-proline-alanine (NPA) sequence and fold back into the membrane where the two NPA sequences align to form a narrow passage at the centre of the channel [12]. A second narrow pore constriction is found towards the apoplastic side of the channel consisting of four amino acid residues of which arginine is highly conserved and often accompanied by the aromatic phenylalanine. This restriction is therefore also referred to as the ar/R selectivity filter and is essential for regulating aquaporin permeability to slightly larger neutral molecules such as urea and glycerol, as well as for the exclusion of protons from the pore [13]. The ar/R selectivity filter, however, does not appear to determine the water or H_2_O_2_ permeability of aquaporins as the residues making up this constriction are identical and yet their permeability to, H_2_O and H_2_O_2_, varies [1, 14, 15]. Furthermore, mutagenesis studies on the ar/R selectivity filter have failed to find evidence for variation in water or H_2_O_2_ permeability and altering the pore diameter at this location appears not to affect water permeability [13]. This leaves the pore constriction at the NPA sequence as the main candidate for determination of aquaporin water permeability. This sequence is also itself highly conserved among all plant PIPs and cannot alone account for differences in PIP water or H_2_O_2_ permeability. Nevertheless, these differences may at least in part be explained by subtle structural effects on the NPA motif [13], which could be brought about during tetramer formation [15–17]. Despite these long-standing unexplained results, there has to date only been one study of H_2_O_2_-permability of PIPs *in planta*, which found that *At*PIP1;4 conducts this molecule, despite the fact that it appeared to be H_2_O_2_-impermeable when expressed in yeast [7].

Plant aquaporins are regulated by various mechanisms that may not be present in yeast or are unable to target plant isoforms [18–20]. Thus, a survey of all PIPs in the fully functional plant environment is required to evaluate the capacity of these aquaporins to facilitate transmembrane H_2_O_2_ diffusion. In this study, we examined various PIP knockout mutants of *A. thaliana* to verify whether their previously determined permeability to H_2_O_2_ has a physiologically relevant role in plant development and water relations.

In addition to permeating certain aquaporins, H_2_O_2_ has also been shown to inhibit water transport through aquaporins when used as a non-specific aquaporin inhibitor in plants due to its low toxicity compared to other inhibitors [21, 22]. However, application of H_2_O_2_ to the root system has also been found to regulate the gene expression of some PIP2 isoforms [1]. Application of exogenous H_2_O_2_ leads to a reduction in root hydraulic conductivity (*L*_pr_) [23–25], which cannot be attributed to its direct effect on aquaporin activity, transcript levels or membrane trafficking, not to mention the regulation of aquaporin activity through phosphorylation or protein internalisation [24, 25]. Our goal was to use a top-down approach to shed light on the effect of exogenous H_2_O_2_ application to the roots on plant development by characterising whole-plant responses to this treatment.

Based on recent studies on the permeability of all 13 *At*PIP isoforms to H_2_O_2_ in yeast (Table 1) [3], and the fact that a reduction in *L*p_r_ in response to exogenous H_2_O_2_ application has been corroborated in multiple studies [23, 24, 26, 27], we expected PIPs to be active in a plant’s response to oxidative stress in *A. thaliana* roots. We, therefore, hypothesized that the growth of knockout mutants lacking PIPs would be less affected by the exogenous H_2_O_2_ treatments than that of the wild type plants due to the reduced H_2_O_2_ influx from the apoplast. We also expected that mutant plants lacking PIP1s or PIP2s would respond differently to the H_2_O_2_ treatments due to their divergent H_2_O_2_ permeabilities (Table 1, [3]). To test this hypothesis, we exposed plant roots to two treatments of contrasting H_2_O_2_ concentrations and examined the effects of these treatments on plant growth and root system architecture. All PIPs investigated in this study and their previously determined H_2_O_2_ permeabilities are summarised in Table 1.

## Results

### Effects on biomass

**Fig. 1:**
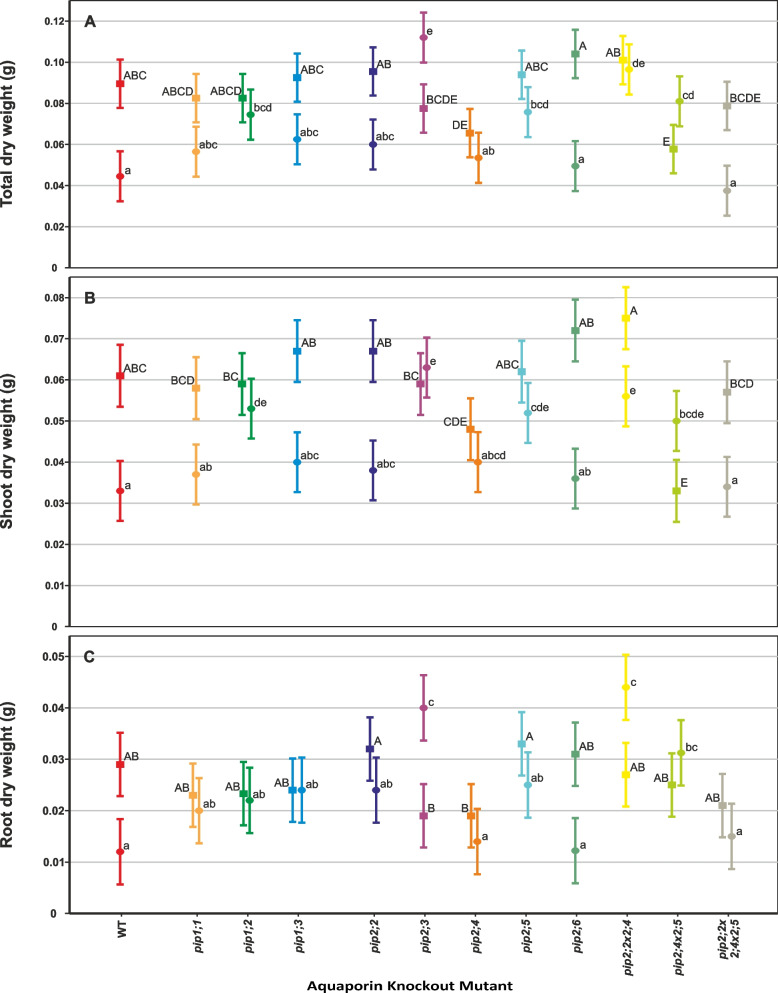
Biomass accumulation by PIP knockout-mutant plants over the course of the experiment. Means ± pooled SE, *n* = 8 – 10 plants. Square symbols denote control conditions and circles treatment with 1 mM H_2_O_2_. Different letters indicate significant differences between the lines under control (upper case) and H_2_O_2_ treatment (lower case) conditions. The relative increase/decrease in biomass due to the treatment is given for each plant line in Table 2. **A** Total dry mass. **B** Shoot dry mass. **C** Root dry mass

**Fig. 2:**
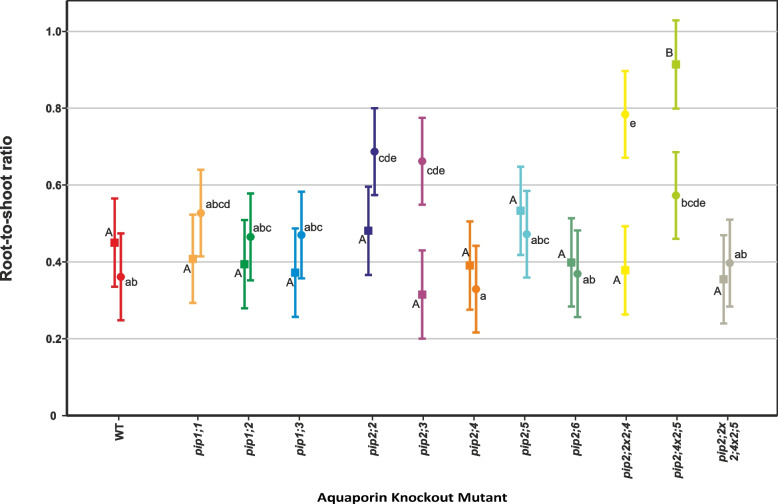
Root-to-shoot ratio of individual plants at 31 days old. Means ± SE, *n* = 8 – 10 plants. Square symbols denote control conditions and circles treatment with 1 mM H_2_O_2_. Different letters indicate significant differences between the lines under control (upper case) and H_2_O_2_ treatment (lower case) conditions. The relative increase/decrease in the ratio due to the treatment is given for each plant line in Table 2

Under controlled conditions, total dry weight (DW) accumulation over the course of the experiment was uniform among most genotypes, but two mutant lines, *pip2;4* and *pip2;4* × *2;5*, had statistically significantly lower DW compared to the wild type (WT) (*p* = 0.045 and 0.011 respectively, Fig. 1A, Table 2). In the case of *pip2;4* × *2;5*, this effect was due to a significantly lower shoot biomass (-46%, *p* < 0.001), which resulted in its root-shoot ratio being twice that of the WT (*p* < 0.001, Fig. 2, Table 2).

**Table 2:** Summary of the H_2_O_2_ treatment effect on plant dry weights and root:shoot ratios. The treatment effects of each parameter are given for each plant line. The *p*-values are listed for mutants that significantly differed from the WT under the treatment

Plant Line	Total Dry Weight	Shoot Dry Weight	Root Dry Weight	Root-Shoot Ratio
WT	-50%	-46%	-59%	-20%
*pip1;1*	-31%	-36%	-13%	+ 29%
*pip1;2*	-10% *p* = 0.016	-10% *p* = 0.007	-4%	+ 18%
*pip1;3*	-32%	-40%	± 0	+ 26%
*pip2;2*	-38%	-43%	-25%	+ 43% *p* = 0.005
*pip2;3*	+ 44% *p* < 0.001	+ 7% *p* < 0.001	+ 111% *p* < 0.001	+ 110% *p* = 0.010
*pip2;4*	-18%	-17%	-26%	-16%
*pip2;5*	-19% *p* = 0.013	-16% *p* = 0.009	-24% *p* = 0.045	-11%
*pip2;6*	-52%	-50%	-61%	-8%
*pip2;2* × *2;4*	-4% *p* < 0.001	-25% *p* = 0.001	+ 63% *p* < 0.001	+ 107% *p* < 0.001
*pip2;4* × *2;5*	+ 40% *p* = 0.006	+ 52% *p* = 0.017	+ 24% *p* = 0.006	-37%
*pip2;2* × *2;4* × *2;5*	-39%	-40%	-29%	+ 12%

The 1 mM H_2_O_2_ treatment caused a 50% reduction of total DW accumulation (shoot + root) in the WT plants, but a much smaller reduction in most of the mutant lines, while *pip2;3* and *pip2;4* × *2;5* even increased their total DW accumulation due to the H_2_O_2_ treatment (Fig. 1A, Table 2). Thus, the following lines accumulated significantly more dry mass than the wild type under the H_2_O_2_ treatment: *pip1;2* (+ 67%, *p* = 0.016), *pip2;3* (+ 149%, *p* < 0.001), *pip2;5* (+ 69%, *p* = 0.013), *pip2;2* × *2;4* (+ 116%, *p* < 0.001) and *pip2;4* × *2;5* (+ 80%, *p* = 0.006). This was largely due to shoot DW, which was also significantly greater compared to the wild type: *pip1;2* (+ 61%, *p* = 0.007), *pip2;3* (+ 91%, *p* < 0.001), *pip2;5* (+ 58%, *p* = 0.009), *pip2;2* × *2;4* (+ 70%, *p* = 0.001) and *pip2;4* × *2;5* (+ 52%, *p* = 0.017) (Fig. 1B, Table 2). With the exception of *pip1;2*, the same set of plant lines also had a longer roots compared to the WT under the treatment, though the contribution of root DW to total DW was smaller: *pip2;3* (+ 233%, *p* < 0.001), *pip2;5* (+ 108%, *p* = 0.045), *pip2;2* × *2;4* (+ 267%, *p* < 0.001) and *pip2;4* × *2;5* (+ 158%, *p* = 0.006) (Fig. 1C). In line with the differential responses of the mutant plants to H_2_O_2_, the root-shoot ratios of the following mutants were significantly higher compared to the wild type in this treatment: *pip2;2* (+ 90%, *p* = 0.005), *pip2;3* (+ 83%, *p* = 0.010) and *pip2;2* × *2;4* (+ 117%, *p* < 0.001).

H_2_O_2_ treatment had a large effect on the wild type with a 46% and 59% reduction in shoot and root DW accumulation, respectively (Table 2). Many of the mutant lines tested here responded differently. However, *pip1;2*, *pip2;4* and *pip2;5*, remained fairly unresponsive to the treatment, while only the shoot DW of *pip2;4* × *2;5* double mutant significantly increased but there was little change in its root DW. Furthermore, the single mutant *pip2;3* displayed an increase of total DW in response to the H_2_O_2_ treatment, which was almost entirely due to greatly enhanced root DW, as is apparent in the two-fold increase of its root-shoot ratio. In fact, *pip2;6* was the only mutant line to have displayed a similar response to the treatment as the wild type in terms of its direction as well as magnitude.

Rates of photosynthesis and stomatal conductance were uniform amongst all plant lines as well as between the control and H_2_O_2_ treatment, and thus there were no significant differences in rates of gas exchange between PIP knockout mutants. Values for the rates of photosynthesis and stomatal conductance are shown in Supplementary Table S1 of Additional file 2.

### Effects on relative (RWC) and absolute (AWC) water content

**Fig. 3:**
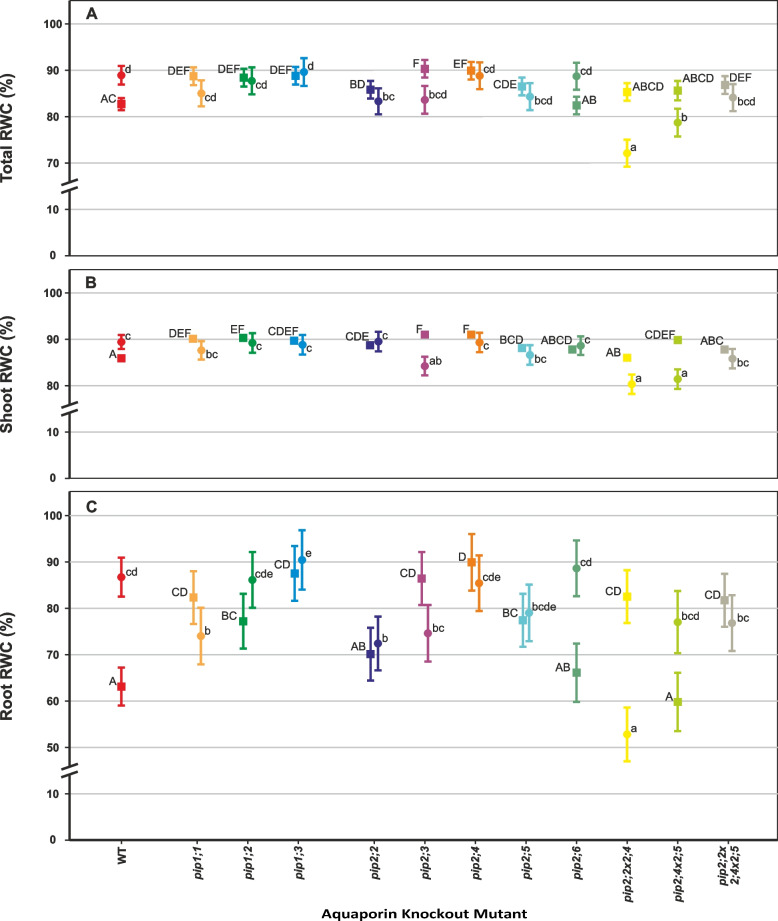
Relative water content (RWC) of individual plants at 31 days old. Means ± SE, *n* = 8 – 10 plants. Square symbols denote control conditions and circles treatment with 1 mM H_2_O_2_. Different letters indicate significant differences between the lines under control (upper case) and H2O2 treatment (lower case) conditions. The relative increase/decrease in RWC due to the treatment is given for each plant line in Table 3. **A** Whole-plant RWC. **B** Shoot RWC. **C** Root RWC

**Fig. 4:**
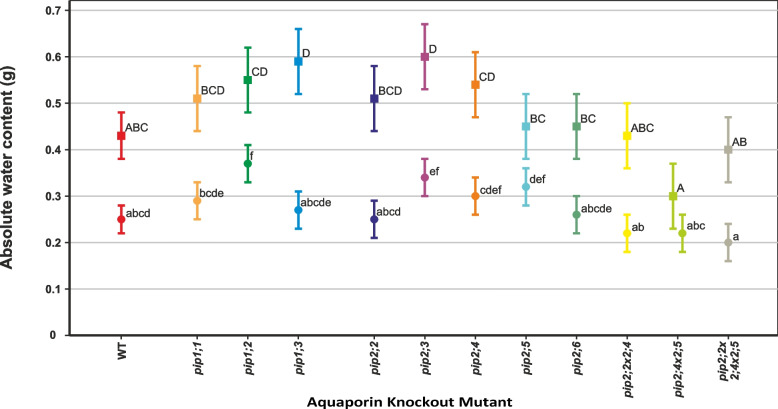
Absolute water content (AWC) of individual rosettes at 31 days old. Means ± SE, *n* = 8 – 10 plants. Square symbols denote control conditions and circles treatment with 1 mM H_2_O_2_. Different letters indicate significant differences between the lines under control (upper case) and H_2_O_2_ treatment (lower case) conditions. The relative decrease in AWC due to the treatment is given for each plant line in Table 3

Under controlled conditions, the relative water content (RWC) of most mutants was significantly higher than the WT in both the roots and shoots (Fig. 3). The exceptions were *pip2;6* that did not differ from the WT; *pip2;2* × *2;4* and *pip2;2* × *2;4* × *2;5* that only had significantly higher root RWC (*p* < 0.001 and *p* = 0.002, respectively), but not shoot RWC. Also, *pip2;2* and *pip2;4* × *2;5*, had increased shoot RWC but not root RWC compared to the WT (*p* = 0.012 and *p* < 0.001, respectively). Absolute water content (AWC) of the shoots was uniform among most genotypes, but significantly higher in *pip1;3* and *pip2;3* compared to the WT (*p* = 0.019 and 0.010 respectively, Fig. 4).

Most of the plant’s water was present in the shoot, therefore the shoot RWC and RWC of the whole plant were very similar, and both changed little in response to the H_2_O_2_ treatment (Fig. 3A and B). However, there were large differences in root RWC between some mutants under control conditions as well as a large effect of the H_2_O_2_ treatment on root RWC in others (Fig. 3C; Table 3). Most notably, compared to the control treatment (0 mM H_2_O_2_), the root RWC was increased in *pip2;6* and *pip2;4* × *2;5* (+ 34% and + 29% respectively), whereas root RWC was reduced in *pip2;2* × *2;4* by 36% in response to the H_2_O_2_ treatment (Fig. 3).

**Table 3:** Summary of the H_2_O_2_ treatment effect on RWC and AWC. The percentage values indicate change in the measured parameters for plants of each line subjected to the 1 mM H_2_O_2_ treatment compared with untreated control. The *p*-values are listed for mutants that significantly differed from the WT under the treatment

Plant Line	Total RWC	Shoot RWC	Root RWC	Shoot AWC
WT	+ 7%	+ 4%	+ 37%	-42%
*pip1;1*	-4%	-3%	-10% *p* = 0.042	-43%
*pip1;2*	-1%	-1%	+ 12%	-33% *p* = 0.003
*pip1;3*	+ 1%	-1%	+ 3%	-54%
*pip2;2*	-3% *p* = 0.050	+ 1%	+ 3% *p* = 0.016	-51%
*pip2;3*	-7%	-7% *p* = 0.012	-14%	-43% *p* = 0.026
*pip2;4*	-1%	-2%	-5%	-44%
*pip2;5*	-3%	-2%	+ 2%	-29%
*pip2;6*	+ 8%	+ 1%	+ 34%	-42%
*pip2;2* × *2;4*	-15% *p* < 0.001	-7% *p* < 0.001	-36% *p* < 0.001	-49%
*pip2;4* × *2;5*	-8% *p* < 0.001	-9% *p* < 0.001	+ 29%	-27%
*pip2;2* × *2;4* × *2;5*	-3%	-2%	-6%	-50%

The AWC of the shoot was reduced in all plant lines in response to the H_2_O_2_ treatment (Fig. 4; Table 3). Of these, *pip1;2* experienced one of the smallest relative reductions and had significantly higher AWC than the WT under the H_2_O_2_ treatment (*p* = 0.003). *pip2;3* had significantly higher AWC than the WT under both conditions (*p* = 0.010 under 0 mM H_2_O_2_ and *p* = 0.026 under 1 mM H_2_O_2_), despite an equivalent relative decrease in response to H_2_O_2_.

**Fig. 5:**
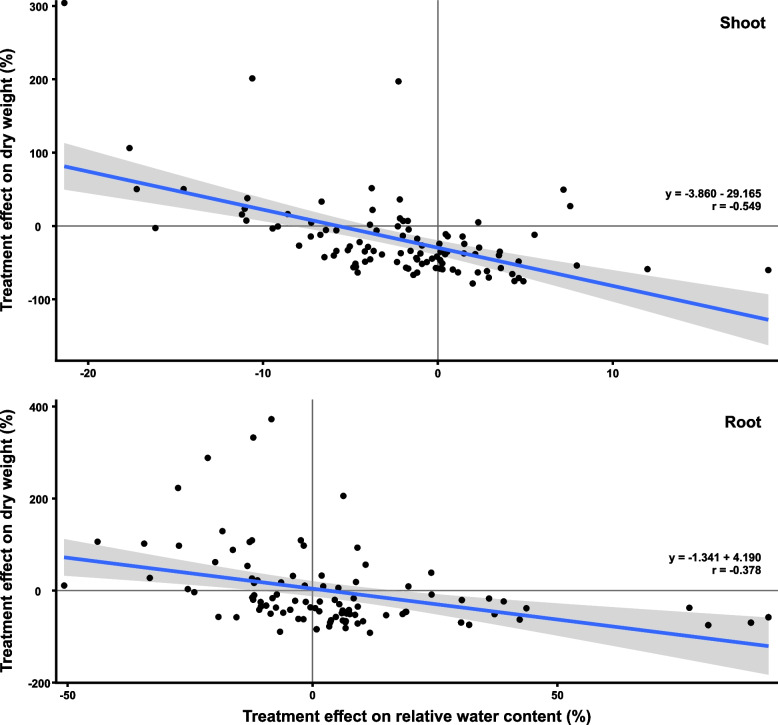
Relationship between the treatment effect on dry mass and RWC. The shaded area indicates the 95% confidence interval. The correlation was statistically significant for both, shoots and roots (*p* < 0.001)

Since we noticed that the change in RWC caused by H_2_O_2_ was in the opposite direction compared to the change in dry weight we tested this relationship (Fig. 5). There was indeed a statistically significant (*p* < 0.001) negative correlation between the H_2_O_2_ treatment effect on dry weight and on RWC (-0.378 in roots and -0.549 in shoots) across all genotypes.

### Effects on root system architecture

**Fig. 6:**
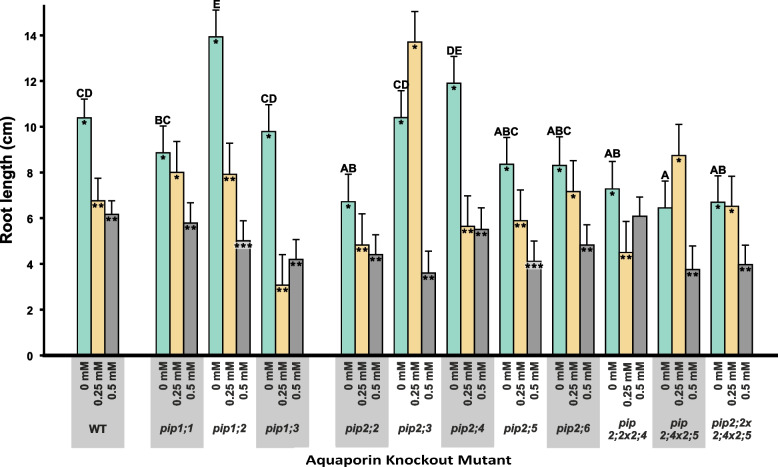
Total root length for all plant lines under three H_2_O_2_ concentrations. Given are means with SE for *n* = 9 – 18. Letters indicate statistically significant differences between the plant lines under control conditions. For reasons of clarity, letters indicating significant differences for the H_2_O_2_ treatments have been omitted from the graph but can be found in Supplementary Table S2 of Additional file 2. Asterisks indicate statistically significant effects of the treatment on a plant line. Different numbers of asterisks indicate a significant effect by the treatment, while columns with no asterisk do not differ from either of the treatments

When grown on MS medium without added H_2_O_2_, only the *pip1;2* mutant had significantly greater root length compared to the WT (+ 34%, *p* = 0.003), while *pip2;2* (-35%, *p* = 0.003), *pip2;2* × *2;4* (-30%, *p* = 0.011), *pip2;4* × *2;5* (-38%, *p* = 0.001) and *pip2;2* × *2;4* × *2;5* (-36%, *p* = 0.002) significantly shorter roots (Fig. 6).

**Fig. 7:**
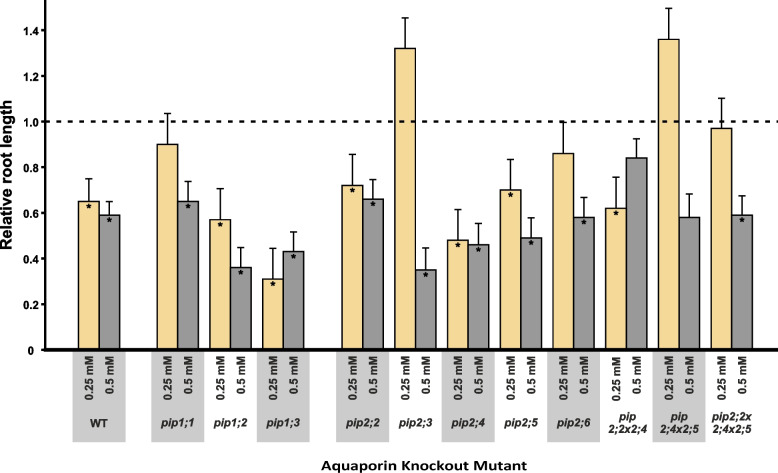
Relative root length of PIP knockout mutants under control and H_2_O_2_ treatments. Root length under control conditions has been set to 100% (indicated by the dashed line). Absolute values for root length can be found in Fig. 6 and Supplementary Table S2 in Additional file 2. Asterisks inside the columns indicate statistically significant treatment effects. Given are means ± SE for *n* = 9 – 18 plants

Almost all the plant lines responded to the H_2_O_2_ treatment with a reduction in root length as shown in Fig. 6, albeit the length and number of lateral roots were affected less by the treatments than total root length (Figure S1 in Additional file 1 and Table S2 in Additional file 2). The WT responded strongly to the H_2_O_2_ treatments displaying a significant reduction in root length by about 40% (*p* < 0.001 at both H_2_O_2_ concentrations, Fig. 6), as did most knockout mutants. However, *pip1;1*, *pip2;5* as well as *pip2;6*, were less responsive to H_2_O_2_, with no significant effect at 0.25 mM H_2_O_2_. The *pip2;2* × *2;4* double-mutant remained unaffected even at 0.5 mM H_2_O_2_ (Fig. 7). The increase in root length in the two mutants, *pip2;3* and *pip2;4* × *2;5*, in response to 0.25 mM H_2_O_2_ was not statistically significant.

### Effects on gene expression

**Fig. 8:**
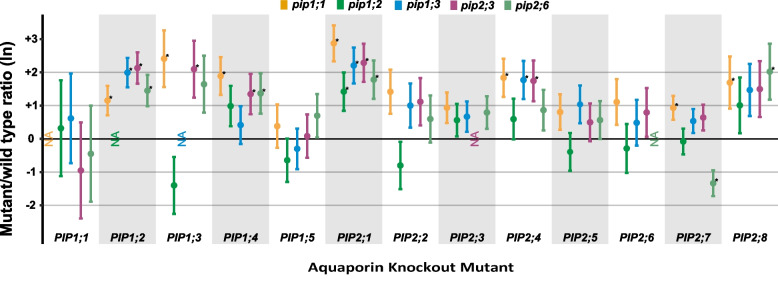
*PIP* gene expression in the mutant lines. Ratios (ln) of *AtPIP* expression levels for *pip1;1*, *pip1;2*, *pip1;3*, *pip2;3* and *pip2;6* knockout mutants. Values are means ± SE for *n* = 4 biological replicates. Asterisks indicate statistically significant differences compared to the WT

Knocking out individual *PIP*s resulted in an overall upregulation of other *PIP*s under standard growing conditions (Fig. 8), which is in line with results previously reported on *PIP* gene expression in *pip2;2*, *pip2;4*, *pip2;5*, *pip2;2* × *2;4*, *pip2;4* × *2;5* and *pip2;2* × *2;4* × *2;5* [28]. This upregulation was most apparent in plant lines lacking aquaporins belonging to the *PIP1* subgroup. For example, *PIP1;2* (*p* = 0.023), *PIP1;3* (*p* = 0.018), *PIP1;4* (*p* = 0.005), *PIP2;1* (*p* < 0.001), *PIP2;4* (*p* = 0.006), *PIP2;7* (p = 0.021) as well as *PIP2;8* (*p* = 0.049) were all significantly upregulated in the *pip1;1* mutant. In *pip1;3*, *PIP1;2* (*p* < 0.001), *PIP2;1* (*p* = 0.001) and *PIP2;4* (*p* = 0.008) were significantly upregulated. Amongst the *PIP2* subgroup, knocking out *PIP2;3* had the highest impact on the expression of other *PIP* genes; causing the significant upregulation of *PIP1;2* (*p* < 0.001), *PIP1;3* (*p* = 0.034), *PIP1;4* (*p* = 0.043), *PIP2;1* (*p* = 0.001) as well as *PIP2;4* (*p* = 0.013). In the *pip2;6* mutant, we found a significant upregulation of *PIP1;2* (*p* = 0.010), *PIP1;4* (*p* = 0.041), *PIP2;1* (*p* = 0.008) and *PIP2;8* (*p* = 0.030) as well as a downregulation *PIP2;7* (*p* = 0.004).

## Discussion

Efficient root water uptake and transport are vital for a plant’s survival and growth. A major factor limiting plant water transport is root hydraulic conductivity (*L*p_r_), which is highly responsive to the expression and activity of PIPs [29–33]. *L*p_r_ as well as PIP expression and activity have been reported to be reduced by H_2_O_2_ treatments in various plant species [1, 24, 27, 34]. This is consistent with the reduced root and shoot biomass accumulation, as well as decreased root length, that we found here and was reported by Claeys et al., 2014 [35] in WT *A. thaliana* plants subject to H_2_O_2_ treatment. Reduced productivity was not accompanied by a change in the rates of gas exchange due to the H_2_O_2_ treatment (Supplementary Table S1 in Additional file 2). We found stomatal conductance to be unresponsive to oxidative stress applied to the roots, which is in agreement with previous observations [26]. This suggests that in our hydroponic set-up *L*p_r_ may not constitute a limiting factor, because water is abundantly available [36] or alternately it may imply that the expression of PIP genes in leaves remains unaffected by H_2_O_2_ application to the roots as reported previously [1]. Thus, the reduced biomass of WT plants is most likely due to impairment of cell expansion by H_2_O_2_ [10]. Reduced cell expansion would also impact leaf area, which we estimated using absolute water content (AWC) as a proxy [37]; allowing us to verify that the H_2_O_2_ treatment reduced shoot growth (Fig. 4).

AWC was significantly increased in *pip1;3* and *pip2;3* under controlled conditions (Fig. 4), wherein both these knockout mutants showed increased expression of *PIP1;2*, *PIP2;1* and *PIP2;4* (Fig. 8); genes which have all been implicated in plant water transport [28, 38–41]. Thus, the increased AWC of these two knock-out mutants may be due to compensatory upregulation of other aquaporins [28]. Nevertheless, the increased shoot growth of *pip2;3* compared to the WT was maintained when grown with H_2_O_2_ (Fig. 4), indicating a certain tolerance of the applied treatment. Furthermore, the H_2_O_2_ treatment only had a modest effect on the root growth of *pip2;3* (Figs. 7), which points to a role of PIP2;3 in facilitating H_2_O_2_ diffusion. Rosette growth is very sensitive to a large range of stress intensities, including mild stress not producing a visible phenotype [35], and thus the fact that AWC of *pip2;3* differed from the WT in the control as well as H_2_O_2_ treatment could indicate an intrinsically higher tolerance of these plants to oxidative stress. However, our current knowledge of the roles of PIP2;3 does not allow for the clear separation of its contributions to cell expansion and growth as opposed to stress signalling. This is an aspect of aquaporin function that will need to be addressed in future studies.

At low concentrations (i.e., 0.01 mM for *A. thaliana* and ≤ 0.5 mM for *Phaseolus vulgaris*), H_2_O_2_ can have a minor stimulatory effect on root growth [24, 27, 42]. This may imply that the absence of a specific H_2_O_2_-permeable PIP *in planta* could impede entry of H_2_O_2,_ limiting its intracellular concentration, and resulting in either a diminished stress response or even a stimulatory effect on growth [24, 27]. This was the case for *pip2;3* and *pip2;4* × *2;5* in terms of their increased root length at 0.25 mM H_2_O_2_ (Fig. 7). The differential results we obtained at different H_2_O_2_ concentrations suggest that there is a threshold concentration at which H_2_O_2_ switches from constituting a potentially stimulatory signal to a stressor [24, 27], and that this threshold changes according to the specific PIPs expressed in the roots. Both *pip2;3* and *pip2;4* × *2;5* had higher root, as well as shoot, biomass at 1 mM H_2_O_2_ (Fig. 1). We, therefore, argue that the lack of functional PIP2;3 and, both, PIP2;4 and PIP2;5 reduces plasma membrane permeability to H_2_O_2,_ allowing only a non-inhibitory amount of H_2_O_2_ to enter root cells at the concentrations used in our treatments. However, confirmation of this hypothesis will require the direct measurement of intracellular levels of H_2_O_2_ concentrations.

A plant’s response under stress conditions is not only determined by the stress itself, but also that plant’s tolerance of the stress [35, 42]. The lack of PIPs permeable to H_2_O_2_ should enhance stress tolerance and, thus, knockout mutants would be expected to display a less pronounced response to H_2_O_2_ compared to the WT. At 0.25 mM H_2_O_2_, root length was unresponsive to H_2_O_2_ in *pip1;1*, *pip2;6* and *pip2;2* × *2;4* × *2;5*, whereas similarly to the WT, *pip2;2* × *2;4* responded by decreasing root growth but was far less responsive at 0.5 mM H_2_O_2_ (Fig. 7). We see this as further evidence that reduced PIP expression lowers the plasma membrane’s permeability to H_2_O_2_ in the roots and raises plants’ resistance to, or perception of, oxidative stress. It furthermore points to non-redundant roles of aquaporins in facilitating H_2_O_2_ diffusion and in stress signalling.

The accumulation of DW was reduced by the H_2_O_2_ treatment in most plant lines [10, 35], but interestingly, this treatment effect was accompanied by an increase in RWC (Fig. 3). In the roots, one possible explanation for this correlation would be that root volume remained constant despite the reduction in DW caused by the treatment. We found the H_2_O_2_ treatment to cause a significant reduction in root length in all plant lines (Fig. 7).

Comparing the PIP knockout mutants to the WT under control conditions, we found some differences that are indicative of the, perhaps overlapping, but nevertheless non-redundant roles of plasma membrane aquaporins. For example, *pip1;2* had significantly longer roots (Fig. 6), which supports previous results by Kaldenhoff et al. [30] who observed 5-times larger root systems (in terms of fresh weight) in *PIP1;1*/*PIP1;2* antisense lines. Notably, in the present work, root dry mass was not altered in *pip1;2*, but instead root RWC was significantly higher than that of the WT (Figs. 1 and 3) and AWC was significantly increased under the H_2_O_2_ treatment (Fig. 4). Significantly decreased root length compared to the WT was recorded in *pip2;2*, *pip2;2* × *2;4*, *pip2;4* × *2;5* and *pip2;2* × *2;4* × *2,5* (Fig. 6); a set which includes all mutant lines lacking functional *PIP2;2*. *PIP2;2* is abundantly expressed in roots [25, 29, 32, 33, 41] and has been found to contribute to lateral root emergence [40] as well as hydraulic conductivity in cortex cells [29]. It is thus not surprising, that the absence of functional *PIP2;2* has a detrimental effect on root development and growth. Interestingly, despite its abundant expression in the plant, non-functional *PIP2;2* does not cause the upregulation of other *PIP* genes [28, 29]. Increased root length and thus a larger surface area for water absorption could effectively compensate for diminished water uptake due to the lack of functional PIPs, supporting past reports that PIP-type aquaporins facilitate root water uptake [29–31, 33, 40], while at the same time providing an explanation for why greenhouse-grown PIP knockout mutants do not display visible phenotypes [28–30, 43].

We found that the RWC was higher in most of our mutant lines than in the WT under control conditions (Fig. 3). Though counterintuitive, as one might expect that a lower RWC in plants would be indicative of disrupted water uptake and translocation, our results could be explained by compensatory upregulation of other PIPs (Fig. 8, [28]). However, clear compensatory upregulation was not present in all knockout mutants (e.g., *pip1;*2, *pip2;4*, *pip2;*5, *pip2;6*) despite their significantly elevated RWC. Furthermore, changes in PIP expression in response to the lack of another isoform were only modest [28]. This suggests that the role of individual PIPs in regulating RWC may be relatively minor, but to establish this would require further research into its significance and specificity among plants.

## Conclusion

Using knockout mutants lacking specific plasma membrane aquaporins, we were able to show that PIP1;1, PIP2;3 and PIP2;6 are permeable to H_2_O_2_ *in planta* and that transmembrane diffusion of H_2_O_2_ plays a physiologically relevant role in plant responses to oxidative stress. We found that PIP2;2 is involved in the regulation of root growth, specifically root length in *A. thaliana*. Since PIPs are physiologically relevant conduits for H_2_O_2_ diffusion into root cells, they are implicated in regulating the effects of H_2_O_2_ on plant growth. Further clarification of the roles of PIPs in H_2_O_2_ signalling and stress responses will require precise measurements of intracellular H_2_O_2_ concentrations as well as a better understanding of how PIP knockout mutations impact plant development.

## Materials and methods

### Plant material and hydrogen peroxide treatment

Seeds for the following single knock-out T-DNA mutants were obtained from the Nottingham Arabidopsis Stock Centre (NASC – www.arabidopsis.org): PIP1;1 (N590778), PIP1;2 (N657533), PIP1;3 (N551107), PIP2;2 (N871747), PIP2;3 (N617876), PIP2;4 (N105980), PIP2;5 (N117303) and PIP2;6 (N573519). The correct T-DNA insertion of all plant lines was confirmed by PCR using the primers listed in Supplementary Table S3 of Additional file 2 and only plants homozygous for the knock-out mutation were used to produce a seeds stock. Double and triple mutants (*pip2;2* × *2;4*, *pip2;4* × *2;5*, *pip2;2* × *2;4* × *2;5*) were created by crossing and homozygosity confirmed by PCR [28].

Seeds were sown in horticultural soil and kept at 4 °C for four days before being transferred to a controlled-environment growth room with a photoperiod of 12 h, photosynthetically active radiation 350 µmol m^−2^ s^−1^ (Philips 86 W 96in T8 High Output Neutral White Fluorescent Tube, F96T8/TL835/HO/PLUS ALTO, USA), 23 °C/18 °C day/night temperatures and ≈30% daytime relative humidity. Three days after germination, seedlings were transplanted into 8 × 8 cm pots (one plant/pot) and grown for another 10 days before washing their roots and transferring to a hydroponic system in the same growth room. The hydroponic system consisted of 4 L-containers with aerated nutrient solution containing 1.25 mM KNO_3_, 1.5 mM Ca(NO_3_)_2_, 0.75 mM MgSO_4_, 0.5 mM KH_2_PO_4_, 50 mM H_3_BO_3_, 10 mM MnCl, 2 mM ZnSO_4_, 1.5 mM CuSO_4_, 75 µM (NH_4_)_2_MoO_4_, and 74 mM Fe-EDTA. The solution was renewed every three days.

To begin the treatment, H_2_O_2_ was applied to the nutrient solution to yield a final concentration of 1 mM H_2_O_2_. An exogenous concentration of 1 mM H_2_O_2_ has been reported to inhibit *A. thaliana* growth [35] and was thus chosen as the upper limit for all our experiments. It has to be noted that H_2_O_2_ is unstable and degrades, resulting in lower average concentrations over the course of the treatment [27]. Plants designated for biomass measurements (8–10 plants per line and treatment) were three weeks old at the beginning of the H_2_O_2_ treatment and were harvested nine days later. Gas exchange measurements required slightly larger plants (6 per line and treatment) and, therefore, these measurements were carried out with 31-day-old plants treated with 1 mM H_2_O_2_ for one and three days.

For root system analysis, the plants were grown on square petri dishes containing 0.7% agarose supplemented with full-strength Murashige and Skoog medium (MS) [44] and 1,5% sucrose. For the treatment, H_2_O_2_ was added into the agar medium to yield final concentrations of 0.25 mM and 0.5 mM. These concentrations were chosen based on a preliminary experiment (not included in the results presented here) during which we observed that at concentrations of 0.75 mM and above, root growth ceased entirely for all plant lines. Seeds of all plant lines were first germinated on agarose without H_2_O_2_ after stratification at 4 °C for 3 days. Three days after germination, 9 – 18 seedlings were transferred to H_2_O_2_-containing growth medium (treatment) or H_2_O_2_-free growth medium (control). The root system was scanned using a flat-bed scanner after 10 days of treatment and the images analysed with RootReader2D software [45] (http://www.plantmineralnutrition.net/software/rootreader2d/downloads/index.html).

### Measurements of biomass and water content

Fresh weights, turgid weights, dry weights, and root:shoot (R:S) dry weight ratios were measured in plants growing in hydroponics and treated for 9 days with 1 mM H_2_O_2_. After drying the roots gently with paper towels, roots and shoots were weighed separately to obtain their respective fresh weights. Turgid weights were obtained after floating the shoots and roots on water overnight. They were then dried at 60 °C for two days and re-weighed to obtain their dry weights.

Relative water content was calculated separately for roots, shoots, and whole plants using the following formula:

$$RWC=100*\frac{FW-DW}{TW- DW} <span class='reftype'>(1)</span>$$

where FW denotes fresh weight, DW dry weight and TW turgid weight.

Absolute water content (AWC) has been found to be linearly correlated with the leaf area under various treatments even when leaf morphology was altered [37] and was thus used as a proxy for leaf area in this study:

$$AWC=FW-DW <span class='reftype'>(2)</span>$$

All numeric values for measurements of biomass and water content as well as statistically significant differences between mutant plants and treatments are shown in Supplementary Table S4 of Additional file 2.

### Gene expression

Transcript abundance was measured by qRT-PCR for *pip1;1*, *pip1;2*, *pip1;3*, *pip2;3* and *pip2;6* grown under ideal conditions. The gene expression for the remaining plant lines used in this study has been reported earlier for the same growing conditions [28]. Twelve rosettes per genotype were harvested and immediately frozen in liquid nitrogen. For RNA extraction, three samples of the same genotype were combined and treated together, resulting in *n* = 4. RNA was extracted using the GeneJET Plant RNA Purification Mini Kit (ThermoFisher Scientific) according to the manufacturer’s instructions with the exception that the Plant RNA Lysis Solution was supplemented with β–mercaptoethanol instead of DTT. The quality and concentration of the extracted RNA was determined with an ND-1000 Spectrophotometer (ThermoFisher Scientific) and 1 µg of RNA was used for cDNA synthesis following DNase1 treatment. Maxima H Minus Reverse Transcriptase, oligo(dT) 19 and dNTP (ThermoFisher Scientific) were used in a 30 µl reaction volume for cDNA synthesis, which was then diluted to a final volume of 70 µl. 1 μl of cDNA was used for PCR in triplicate with 5 × HOT FIREPol EvaGreen qPCR Mix Plus (Solis BioDyne, Tartu, Estonia) using a CFX 384 Real-Time PCR detection system (Bio-Rad, Hercules, CA, USA). PIP-specific primers were taken from Alexandersson et al. 2010 [46]. Ct values were converted using the ΔΔCt-method using all three reference genes listed in Table S5 of Additional file 2 and ln-transformed for statistical analysis.

### Gas exchange measurements

Leaf-level gas exchange was measured for one leaf per plant using the portable photosynthesis system LI6400XT infra-red gas analyser (IGRA) equipped with a fluorescence chamber (LI-COR Biosciences, Nebraska, USA). The leaf area covering the chamber window was calculated as described in Israel et al. [28]. Measurements were carried out one and three days after the application of the H_2_O_2_ treatment, when plants were 32 and 34 days old respectively. On each measurement day, a total of six replicate plants were measured for every plant line and treatment. The following settings were used during all measurements: flow 300 µmol s^−1^, Tblock 25 °C, PAR 1500 µmol m^−2^ s^−1^ (10% blue), leaf fan fast, CO2R 400 µmol mol^−1^.

### Data analysis

ANOVAs were conducted in R (package Deducer) using a linear model with plant genotype and the measured variable as the factors to compare the means of all measured variables for the mutant lines to the WT. The number of replicates was *n* = 4 for gene expression analysis, *n* = 6 for gas exchange measurements, *n* = 9 – 18 for root system architecture and *n* = 8 – 10 for biomass and water content measurements.

The correlation between the treatment effect on dry mass and RWC was carried out in R using two-sided Pearson’s correlation with 95% confidence interval. The number of replicates included in the correlation was *n* = 97 for roots and *n* = 101 for shoots.

## Supplementary Information

**Additional file 1:** **Supplementary Figure S1.** Root system architecture. Given are means with SE for *n* = 9 - 18. Letters indicate statistically significant differences betwween the plant lines under control conditions. For reasons of clarity, letters indicating significant differences for the H_2_O_2_ treatments have been omitted from the graph but can be found in **Supplementary Table S2.** Asterisk indicate statiscally significant effects of the treatment on a plant line. Different numbers of asterisks indicate a significant effect by the treatment, while columns with no asterisk do not differ from either of the treatments. A) Total root length (taller and light columns) and primary root length (shorter and darker columns) with SE for all lines and treatments. Total as well as primary root length were measured as the growth after the onset of the treatment. B) Length of secondary roots (taller and lighter columns) and tertiary roots (shorter and darker columns) SE for all lines and treatments. C) Number of secondary roots (taller and lighter columns) tertiary roots (shorter and darker columns) for all lines and treatments with SE.

**Additional file 2:** **Table S1.** Gas exchange, means with SE, in bold significant differences compared to WT. **TableS2.** Root system architecture, means with SE, in bold significant differences compared to WT. **Table S3.** Primers used to genotype the stock lines obtained from NASC. **Table S4.** Biomass and water content, means with SE, in bold significant differences compared to WT. **Table S5.** Primers for PIP and reference genes used in the qRT-PCR.

## Data Availability

All data generated or analysed during this study are included in this published article and its supplementary information files.

## Abbreviations

ar/R: Aromatic/arginine
AWC: Absolute water content
DW: Dry weight
FW: Fresh weight
*L*p_r_: Root hydraulic conductivity
PAR: Photosynthetically active radiation
PIP: Plasma membrane intrinsic protein
qRT-PCR: Quantitative real-time PCR
RWC: Relative water content
WT: Wild type

## References

[1] Hooijmaijers C, Rhee JY, Kwak KJ, Chung GC, Horie T, Katsuhara M (2012). Hydrogen peroxide permeability of plasma membrane aquaporins of Arabidopsis thaliana. J Plant Res.

[2] Dynowski M, Schaaf G, Loque D, Moran O, Ludewig U (2008). Plant plasma membrane water channels conduct the signalling molecule H2O2. Biochem J.

[3] Groszmann M, De Rosa A, Chen W, Qiu J, McGaughey SA, Byrt CS, et al. Permeability profiling of all 13 Arabidopsis PIP aquaporins using a high throughput yeast approach. BioRXiv. 2021. 10.3389/fpls.2023.1078220 PMC9907170 36760647

[4] Kaurilind E, Xu E, Brosche M (2015). A genetic framework for H2O2 induced cell death in Arabidopsis thaliana. BMC Genomics.

[5] Peleg-Grossman S, Volpin H, Levine A (2007). Root hair curling and Rhizobium infection in Medicago truncatula are mediated by phosphatidylinositide-regulated endocytosis and reactive oxygen species. J Exp Bot.

[6] Yao Y, Liu X, Li Z, Ma X, Rennenberg H, Wang X (2013). Drought-induced H2O2 accumulation in subsidiary cells is involved in regulatory signaling of stomatal closure in maize leaves. Planta.

[7] Tian S, Wang X, Li P, Wang H, Ji H, Xie J (2016). Plant Aquaporin AtPIP1;4 Links Apoplastic H2O2 Induction to Disease Immunity Pathways. Plant Physiol.

[8] Bienert GP, Chaumont F (2014). Aquaporin-facilitated transmembrane diffusion of hydrogen peroxide. Bba-Gen Subjects.

[9] Bienert GP, Moller AL, Kristiansen KA, Schulz A, Moller IM, Schjoerring JK (2007). Specific aquaporins facilitate the diffusion of hydrogen peroxide across membranes. J Biol Chem.

[10] Smirnoff N, Arnaud D (2019). Hydrogen peroxide metabolism and functions in plants. New Phytol.

[11] Wang H, Schoebel S, Schmitz F, Dong H, Hedfalk K (2020). Characterization of aquaporin-driven hydrogen peroxide transport. Biochim Biophys Acta Biomembr.

[12] Tornroth-Horsefield S, Hedfalk K, Fischer G, Lindkvist-Petersson K, Neutze R (2010). Structural insights into eukaryotic aquaporin regulation. Febs Lett.

[13] Kitchen P, Salman MM, Pickel SU, Jennings J, Tornroth-Horsefield S, Conner MT (2019). Water channel pore size determines exclusion properties but not solute selectivity. Sci Rep.

[14] Wallace  IS, Roberts  DM (2004). Homology modeling of representative subfamilies of Arabidopsis major intrinsic proteins. Classification based on the aromatic/arginine selectivity filter. Plant Physiol.

[15] Berny MC, Gilis D, Rooman M, Chaumont F (2016). Single mutations in the transmembrane domains of maize plasma membrane aquaporins affect the activity of monomers within a heterotetramer. Mol Plant.

[16] Zelazny E, Borst JW, Muylaert M, Batoko H, Hemminga MA, Chaumont F (2007). FRET imaging in living maize cells reveals that plasma membrane aquaporins interact to regulate their subcellular localization. P Natl Acad Sci USA.

[17] Otto B, Uehlein N, Sdorra S, Fischer M, Ayaz M, Belastegui-Macadam X (2010). Aquaporin tetramer composition modifies the function of tobacco aquaporins. J Biol Chem.

[18] Maurel C (2007). Plant aquaporins: novel functions and regulation properties. Febs Lett.

[19] Santoni V, Verdoucq L, Sommerer N, Vinh J, Pflieger D, Maurel C (2006). Methylation of aquaporins in plant plasma membrane. Biochem J.

[20] Johansson I, Karlsson M, Shukla VK, Chrispeels MJ, Larsson C, Kjellbom P (1998). Water transport activity of the plasma membrane aquaporin PM28A is regulated by phosphorylation. Plant Cell.

[21] Gambetta GA, Fei J, Rost TL, Knipfer T, Matthews MA, Shackel KA (2013). Water uptake along the length of grapevine fine roots: developmental anatomy, tissue-specific aquaporin expression, and pathways of water transport. Plant Physiol.

[22] Kim YX, Steudle E (2009). Gating of aquaporins by light and reactive oxygen species in leaf parenchyma cells of the midrib of Zea mays. J Exp Bot.

[23] Lee SH, Singh AP, Chung GC (2004). Rapid accumulation of hydrogen peroxide in cucumber roots due to exposure to low temperature appears to mediate decreases in water transport. J Exp Bot.

[24] Boursiac Y, Boudet J, Postaire O, Luu DT, Tournaire-Roux C, Maurel C (2008). Stimulus-induced downregulation of root water transport involves reactive oxygen species-activated cell signalling and plasma membrane intrinsic protein internalization. Plant J.

[25] Prak S, Hem S, Boudet J, Viennois G, Sommerer N, Rossignol M (2008). Multiple phosphorylations in the C-terminal tail of plant plasma membrane aquaporins: role in subcellular trafficking of AtPIP2;1 in response to salt stress. Mol Cell Proteomics.

[26] Ehlert C, Maurel C, Tardieu F, Simonneau T (2009). Aquaporin-mediated reduction in maize root hydraulic conductivity impacts cell turgor and leaf elongation even without changing transpiration. Plant Physiol.

[27] Benabdellah K, Ruiz-Lozano JM, Aroca R (2009). Hydrogen peroxide effects on root hydraulic properties and plasma membrane aquaporin regulation in Phaseolus vulgaris. Plant Mol Biol.

[28] Israel D, Khan S, Warren CR, Zwiazek JJ, Robson TM (2021). The contribution of PIP2-type aquaporins to photosynthetic response to increased vapour pressure deficit. J Exp Bot.

[29] Javot H, Lauvergeat V, Santoni V, Martin-Laurent F, Guclu J, Vinh J (2003). Role of a single aquaporin isoform in root water uptake. Plant Cell.

[30] Kaldenhoff R, Grote K, Zhu JJ, Zimmermann U (1998). Significance of plasmalemma aquaporins for water-transport in Arabidopsis thaliana. Plant J.

[31] Lopez F, Bousser A, Sissoeff I, Gaspar M, Lachaise B, Hoarau J (2003). Diurnal regulation of water transport and aquaporin gene expression in maize roots: contribution of PIP2 proteins. Plant Cell Physiol.

[32] Boursiac Y, Chen S, Luu DT, Sorieul M, van den Dries N, Maurel C (2005). Early effects of salinity on water transport in Arabidopsis roots. molecular and cellular features of aquaporin expression. Plant Physiol.

[33] Takase T, Ishikawa H, Murakami H, Kikuchi J, Sato-Nara K, Suzuki H (2011). The circadian clock modulates water dynamics and aquaporin expression in Arabidopsis roots. Plant Cell Physiol.

[34] Wudick MM, Li X, Valentini V, Geldner N, Chory J, Lin J (2015). Subcellular redistribution of root aquaporins induced by hydrogen peroxide. Mol Plant.

[35] Claeys H, Van Landeghem S, Dubois M, Maleux K, Inze D (2014). What is stress? dose-response effects in commonly used in vitro stress assays. Plant Physiol.

[36] Zimmermann HM, Steudle E (1998). Apoplastic transport across young maize roots: effect of the exodermis. Planta.

[37] Hughes AP, Cockshull KE, Heath OVS (1970). Leaf area and absolute leaf water content. Ann Bot.

[38] Li L, Wang H, Gago J, Cui H, Qian Z, Kodama N (2015). Harpin Hpa1 Interacts with Aquaporin PIP1;4 to Promote the Substrate Transport and Photosynthesis in Arabidopsis. Sci Rep.

[39] Postaire O, Tournaire-Roux C, Grondin A, Boursiac Y, Morillon R, Schaffner AR (2010). A PIP1 aquaporin contributes to hydrostatic pressure-induced water transport in both the root and rosette of arabidopsis. Plant Physiol.

[40] Peret B, Li G, Zhao J, Band LR, Voss U, Postaire O (2012). Auxin regulates aquaporin function to facilitate lateral root emergence. Nat Cell Biol.

[41] Da Ines O, Graf W, Franck KI, Albert A, Winkler JB, Scherb H (2010). Kinetic analyses of plant water relocation using deuterium as tracer - reduced water flux of Arabidopsis pip2 aquaporin knockout mutants. Plant Biol.

[42] Aroca R, Amodeo G, Fernandez-Illescas S, Herman EM, Chaumont F, Chrispeels MJ (2005). The role of aquaporins and membrane damage in chilling and hydrogen peroxide induced changes in the hydraulic conductance of maize roots. Plant Physiol.

[43] Kromdijk J, Glowacka K, Long SP (2020). Photosynthetic efficiency and mesophyll conductance are unaffected in Arabidopsis thaliana aquaporin knock-out lines. J Exp Bot.

[44] Murashige T, Skoog F (1962). A revised medium for rapid growth and bio assays with tobacco tissue cultures. Physiol Plantarum.

[45] Clark RT, Famoso AN, Zhao K, Shaff JE, Craft EJ, Bustamante CD (2013). High-throughput two-dimensional root system phenotyping platform facilitates genetic analysis of root growth and development. Plant Cell Environ.

[46] Alexandersson E, Danielson JA, Rade J, Moparthi VK, Fontes M, Kjellbom P (2010). Transcriptional regulation of aquaporins in accessions of Arabidopsis in response to drought stress. Plant J.

