# Supplementary material for: Plasma membrane aquaporins of the PIP1 and PIP2 subfamilies facilitate hydrogen peroxide diffusion into plant roots
Source: BMC Plant Biol. 2022 Dec 5;22:566. doi: 10.1186/s12870-022-03962-6 (PMC9721007; doi:10.1186/s12870-022-03962-6)
Supplement: Supplementary file 1 — Additional file 1: Supplementary Figure S1. Root system architecture. Given are means with SE for n = 9 - 18. Letters indicate statistically significant differences betwween the plant lines under control conditions. For reasons of clarity, letters indicating significant differences for the H2O2 treatments have been omitted from the graph but can be found in Supplementary Table S2. Asterisk indicate statiscally significant effects of the treatment on a plant line. Different numbers of asterisks indicate a significant effect by the treatment, while columns with no asterisk do not differ from either of the treatments. A) Total root length (taller and light columns) and primary root length (shorter and darker columns) with SE for all lines and treatments. Total as well as primary root length were measured as the growth after the onset of the treatment. B) Length of secondary roots (taller and lighter columns) and tertiary roots (shorter and darker columns) SE for all lines and treatments. C) Number of secondary roots (taller and lighter columns) tertiary roots (shorter and darker columns) for all lines and treatments with SE. [file 12870_2022_3962_MOESM1_ESM.pdf]

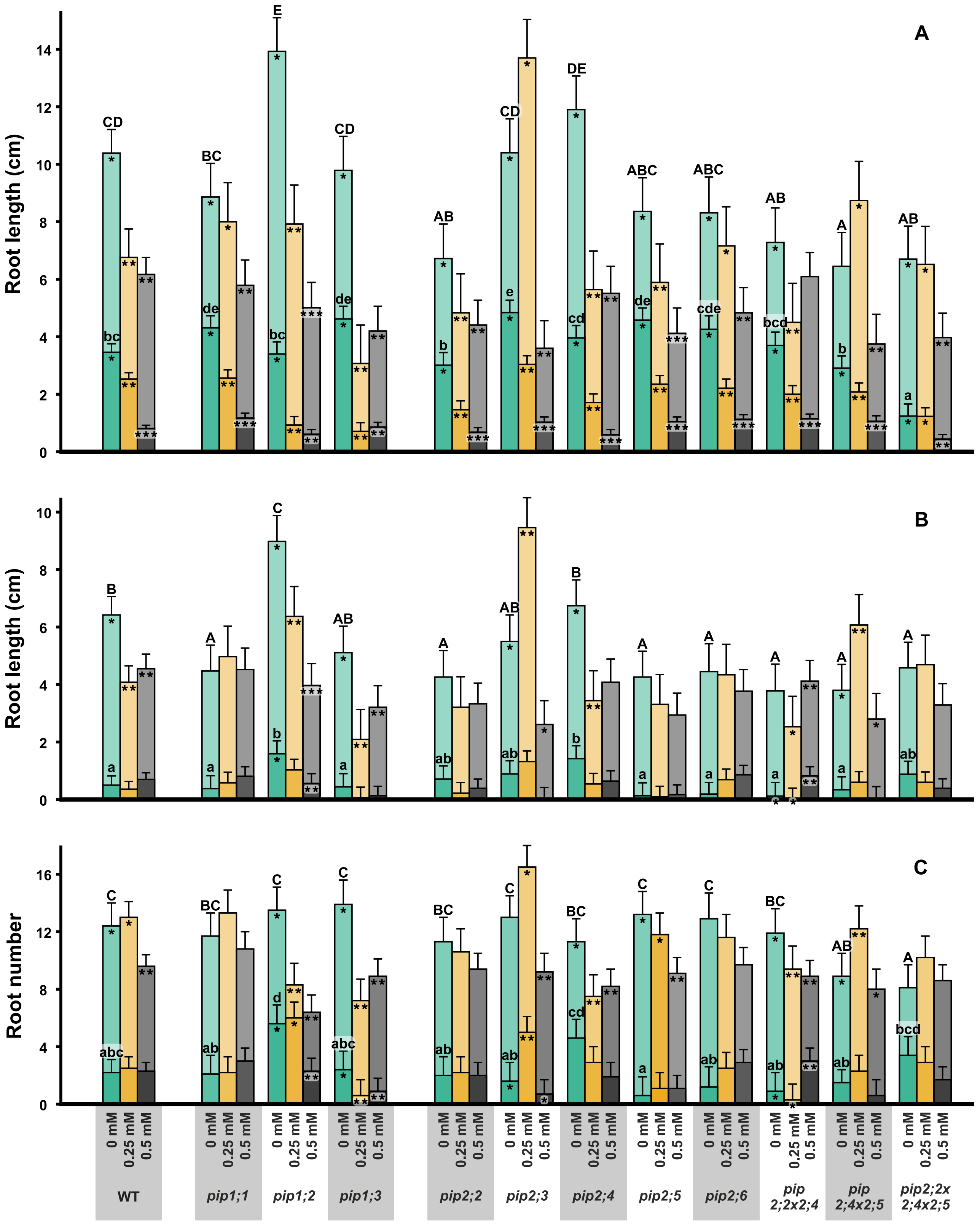

Supplementary Figure S1: Root system architecture. Given are means with SE for n = 9 – 18. Letters indicate statistically significant differences between the plant lines under control conditions. For reasons of clarity, letters indicating significant differences for the H2O2 treatments have been omitted from the graph but can be found in Supplementary Table S2. Asterisks indicate statistically significant effects of the treatment on a plant line. Different numbers of asterisks indicate a significant effect by the treatment, while columns with no asterisk do not differ from either of the treatments. A) Total root length (taller and light columns) and primary root length (shorter and darker columns) with SE for all lines and treatments. Total as well as primary root length were measured as the growth after the onset of the treatment. B) Length of secondary roots (taller and lighter columns) and tertiary roots (shorter and darker columns) SE for all lines and treatments. C) Number of secondary roots (taller and lighter columns) and tertiary roots (shorter and darker columns) for all lines and treatments with SE.
