# Supplementary material for: Plasma membrane aquaporins of the PIP1 and PIP2 subfamilies facilitate hydrogen peroxide diffusion into plant roots
Source: BMC Plant Biol. 2022 Dec 5;22:566. doi: 10.1186/s12870-022-03962-6 (PMC9721007; doi:10.1186/s12870-022-03962-6)
Supplement: Supplementary file 2 — Additional file 2: Table S1. Gas exchange, means with SE, in bold significant differences compared to WT. TableS2. Root system architecture, means with SE, in bold significant differences compared to WT. Table S3. Primers used to genotype the stock lines obtained from NASC. Table S4. Biomass and water content, means with SE, in bold significant differences compared to WT. Table S5. Primers for PIP and reference genes used in the qRT-PCR. [file 12870_2022_3962_MOESM2_ESM.docx]

**Additional File 2**

Table S1: Gas exchange, means with SE, in bold significant differences compared to WT

|  | WT | *pip1;1* | *pip1;2* | *pip1;3* | *pip2;2* | *pip2;3* | *pip2;4* | *pip2;5* | *pip2;6* | *pip2;2x2;4* | *pip2;4x2;5* | *pip2;2x2;4x2;5* |
| --- | --- | --- | --- | --- | --- | --- | --- | --- | --- | --- | --- | --- |
| *A*_net_ control 1d | 7.23 ± 0.70 | 7.18 ± 1.03 | 7.25 ± 0.99 | 7.26 ± 0.99 | 7.68 ± 0.99 | 7.99 ± 0.99 | 6.90 ± 0.99 | 5.80 ± 0.99 | 7.64 ± 0.99 | 7.16 ± 0.99 | 7.00 ± 0.99 | 7.80 ± 0.99 |
| *A*_net_ H_2_O_2_ 1d | 7.22 ± 0.74 | 6.63 ± 1.04 | 6.21 ± 1.09 | 7.40 ± 1.04 | 7.06 ± 1.04 | 7.46 ± 1.04 | 7.69 ± 1.04 | 6.69 ± 1.04 | 6.98 ± 1.04 | 7.02 ± 1.04 | 7.42 ± 1.04 | 7.43 ± 1.04 |
| *g*_s_ control 1d | 0.11 ± 0.02 | 0.13 ± 0.03 | 0.12 ± 0.02 | 0.13 ± 0.02 | 0.15 ± 0.02 | 0.14 ± 0.02 | 0.12 ± 0.02 | 0.09 ± 0.02 | 0.13 ± 0.02 | 0.12 ± 0.02 | 0.12 ± 0.02 | 0.14 ± 0.02 |
| *g*_s_ H_2_O_2_ 1d | 0.13 ± 0.02 | 0.10 ± 0.02 | 0.01 ± 0.03 | 0.12 ± 0.02 | 0.12 ± 0.02 | 0.14 ± 0.02 | 0.13 ± 0.02 | 0.11 ± 0.02 | 0.15 ± 0.02 | 0.13 ± 0.02 | 0.13 ± 0.02 | 0.14 ± 0.02 |
| *A*_net_ control 3d | 7.25 ± 0.63 | 8.20 ± 0.90 | 7.75 ± 0.90 | 7.93 ± 0.90 | 6.99 ± 0.90 | 8.00 ± 0.90 | 7.15 ± 0.90 | 5.85 ± 0.90 | 6.42 ± 0.90 | 6.78 ± 0.90 | 6.26 ± 0.90 | 7.31 ± 0.90 |
| *A*_net_ H_2_O_2_ 3d | 6.69 ± 0.67 | 6.78 ± 0.91 | 6.91 ± 0.99 | 6.85 ± 0.95 | 6.93 ± 0.95 | 6.54 ± 0.95 | 7.40 ± 0.95 | 6.98 ± 0.95 | 7.50 ± 0.95 | 6.38 ± 0.95 | 7.89 ± 0.95 | 6.09 ± 0.95 |
| *g*_s_ control 3d | 0.12 ± 0.02 | 0.14 ± 0.02 | 0.12 ± 0.02 | 0.14 ± 0.02 | 0.14 ± 0.02 | 0.13 ± 0.02 | 0.12 ± 0.02 | 0.09 ± 0.02 | 0.10 ± 0.02 | 0.11 ± 0.02 | 0.09 ± 0.02 | 0.13 ± 0.02 |
| *g*_s_ H_2_O_2_ 3d | 0.12 ± 0.02 | 0.12 ± 0.02 | 0.10 ± 0.02 | 0.13 ± 0.02 | 0.12 ± 0.02 | 0.11 ± 0.02 | 0.12 ± 0.02 | 0.13 ± 0.02 | 0.13 ± 0.02 | 0.11 ± 0.02 | 0.13 ± 0.02 | 0.10 ± 0.02 |

Table S2: Root system architecture, means with SE, in bold significant differences compared to WT

|  | WT | *pip1;1* | *pip1;2* | *pip1;3* | *pip2;2* | *pip2;3* | *pip2;4* | *pip2;5* | *pip2;6* | *pip2;2x2;4* | *pip2;4x2;5* | *pip2;2x2;4x2;5* |
| --- | --- | --- | --- | --- | --- | --- | --- | --- | --- | --- | --- | --- |
| Total root length control (cm) | 10.39 ± 0.82 | 8.86 ± 1.17 | **13.93 ± 1.17 (*p* = 0.003)** | 9.79 ± 1.18 | **6.72 ± 1.20 (*p* = 0.003)** | 10.40 ± 1.18 | 11.90 ± 1.17 | 8.36 ± 1.17 | 8.31 ± 1.25 | **7.28 ± 1.20 (*p* = 0.011)** | **6.45 ± 1.18 (*p* = 0.001)** | **6.70 ± 1.15 (*p* = 0.002)** |
| Total root length 0.25 mM H_2_O_2_ (cm) | 6.76 ± 0.99 | 8.00 ± 1.36 | 7.92 ± 1.36 | **3.07 ± 1.34 (*p* = 0.006)** | 4.83 ± 1.36 | **13.70 ± 1.34 (*p* < 0.001)** | 5.64 ± 1.34 | 5.89 ± 1.34 | 7.16 ± 1.36 | 4.50 ± 1.36 | 8.74 ± 1.36 | 6.52 ± 1.32 |
| Total root length 0.5 mM H_2_O_2_ (cm) | 6.17 ± 0.59 | 5.79 ± 0.88 | 5.01 ± 0.88 | **4.20 ± 0.86 (*p* = 0.024)** | **4.41 ± 0.86 (*p* = 0.043)** | **3.60 ± 0.96 (*p* = 0.008)** | 5.51 ± 0.94 | **4.12 ± 0.88 (*p* = 0.021)** | 4.83 ± 0.88 | 6.09 ± 0.84 | **3.75 ± 1.03 (*p* = 0.019)** | **3.97 ± 0.85 (*p* = 0.010)** |
| Primary root length 0 mM H_2_O_2_ (cm) | 3.46 ± 0.30 | **4.31 ± 0.42 (*p* = 0.046)** | 3.46 ± 0.42 | **4.62 ± 0.44 (*p* = 0.010)** | 3.01 ± 0.44 | **4.84 ± 0.43 (*p* = 0.002)** | 3.96 ± 0.43 | **4.58 ± 0.42 (*p* = 0.009)** | 4.26 ± 0.47 | 3.70 ± 0.46 | 2.91 ± 0.42 | **1.24 ± 0.42 (*p* < 0.001)** |
| Primary root length 0.25 mM H_2_O_2_ (cm) | 2.53 ± 0.22 | 2.56 ± 0.31 | **0.93 ± 0.30 (*p* < 0.001)** | **0.71 ± 0.30 (*p* < 0.001)** | **1.46 ± 0.31 (*p* < 0.001)** | 3.04 ± 0.30 | **1.71 ± 0.30 (*p* = 0.007)** | 2.55 ± 0.30 | 2.21 ± 0.32 | 2.00 ± 0.30 | 2.08 ± 0.31 | **1.23 ± 0.30 (*p* < 0.001)** |
| Primary root length 0.5 mM H_2_O_2_ (cm) | 0.81 ± 0.11 | **1.17 ± 0.17 (*p* = 0.037)** | 0.61 ± 0.16 | 0.86 ± 0.16 | 0.68 ± 0.16 | 1.03 ± 0.18 | 0.79 ± 0.18 | 1.05 ± 0.16 | 1.13 ± 0.16 | **1.15 ± 0.16 (*p* = 0.035)** | 1.06 ± 0.19 | **0.44 ± 0.16 (*p* = 0.025)** |
| Lateral root length 0 mM H_2_O_2_ | 6.92 ± 0.88 | 4.92 ± 1.25 | **10.48 ± 1.25 (*p* = 0.005)** | 5.93 ± 1.27 | 5.01 ± 1.27 | 6.39 ± 1.27 | 8.16 ± 1.25 | **4.39 ± 1.25 (*p* = 0.043)** | 4.64 ± 1.34 | **3.95 ± 1.29 (*p* = 0.022)** | **4.14 ± 1.25 (*p* = 0.027)** | 5.46 ± 1.23 |
| Lateral root length 0.25 mM H_2_O_2_ | 4.52 ± 0.96 | 5.55 ± 1.31 | **7.39 ± 1.29 (*p* = 0.027)** | 2.45 ± 1.29 | 3.43 ± 1.31 | **10.78 ± 1.29 (*p* < 0.001)** | 3.98 ± 1.29 | 3.40 ± 1.29 | 5.03 ± 1.31 | 2.55 ± 1.31 | 6.67 ± 1.31 | 5.29 ± 1.27 |
| Lateral root length 0.5 mM H_2_O_2_ | 5.49 ± 0.68 | 5.39 ± 0.99 | 4.55 ± 1.00 | **3.34 ± 0.99 (*p* = 0.030)** | 3.72 ± 0.79 | **2.66 ± 1.10 (*p* = 0.010)** | 4.72 ± 1.07 | **3.11 ± 1.00 (*p* = 0.018)** | 4.63 ± 0.99 | 4.94 ± 0.96 | **2.90 ± 1.17 (*p* = 0.028)** | 3.68 ± 0.97 |
| Secondary root length 0 mM H_2_O_2_ | 6.42 ± 0.64 | **4.47 ± 0.90 (*p* = 0.032)** | **8.98 ± 0.90 (*p* = 0.005)** | 5.11 ± 0.92 | **4.26 ± 0.92 (*p* = 0.020)** | 5.19 ± 0.92 | 6.74 ± 0.90 | **3.92 ± 0.90 (*p* = 0.006)** | **4.29 ± 0.97 (*p* = 0.030)** | **3.78 ± 0.93 (*p* = 0.005)** | **3.78 ± 0.90 (*p* = 0.004)** | **4.58 ± 0.89 (*p* = 0.041)** |
| Secondary root length 0.25 mM H_2_O_2_ | 4.08 ± 0.77 | 4.97 ± 1.06 | **6.37 ± 1.04 (*p* = 0.030)** | 2.09 ± 1.04 | 3.22 ± 1.06 | **9.43 ± 1.04 (*p* < 0.001)** | 3.43 ± 1.04 | 3.31 ± 1.04 | 4.32 ± 1.06 | 2.53 ± 1.06 | 6.07 ± 1.06 | 4.69 ± 1.03 |
| Secondary root length 0.5 mM H_2_O_2_ | 4.55 ± 0.51 | 4.52 ± 0.75 | 3.97 ± 0.76 | 3.21 ± 0.75 | 3.63 ± 0.72 | **2.61 ± 0.83 (*p* = 0.021)** | 4.07 ± 0.81 | **2.94 ± 0.76 (*p* = 0.036)** | 3.71 ± 0.75 | 4.12 ± 0.72 | 2.80 ± 0.89 | 3.41 ± 0.74 |
| Tertiary root length 0 mM H_2_O_2_ | 0.50 ± 0.32 | 0.38 ± 0.45 | **1.59 ± 0.45 (*p* = 0.017)** | 0.44 ± 0.46 | 0.71 ± 0.46 | 0.89 ± 0.46 | **1.42 ± 0.45 (*p* = 0.045)** | 0.13 ± 0.45 | 0.19 ± 0.49 | 0.12 ± 0.47 | 0.34 ± 0.45 | 0.88 ± 0.45 |
| Tertiary root length 0.25 mM H_2_O_2_ | 0.36 ± 0.27 | 0.58 ± 0.37 | 1.03 ± 0.37 | 0.06 ± 0.37 | 0.22 ± 0.37 | **1.32 ± 0.37 (*p* = 0.009)** | 0.54 ± 0.37 | 0.09 ± 0.37 | 0.69 ± 0.37 | 0.01 ± 0.37 | 0.60 ± 0.37 | 0.60 ± 0.36 |
| Tertiary root length 0.5 mM H_2_O_2_ | 0.70 ± 0.23 | 0.81 ± 0.33 | 0.56 ± 0.34 | 0.13 ± 0.33 | 0.39 ± 0.32 | 0.05 ± 0.37 | 0.64 ± 0.36 | 0.17 ± 0.34 | 0.86 ± 0.33 | 0.82 ± 0.32 | 0.05 ± 0.40 | 0.39 ± 0.33 |
| Lateral root count 0 mM H_2_O_2_ | 14.3 ± 1.4 | 13.5 ± 2.0 | **19.2 ± 2.0 (*p* = 0.018)** | 16.3 ± 2.1 | 13.4 ± 2.1 | 14.9 ± 2.1 | 15.9 ± 2.0 | 13.8 ± 2.0 | 14.2 ± 2.2 | 12.7 ± 2.1 | 10.5 ± 2.0 | 11.5 ± 2.0 |
| Lateral root count 0.25 mM H_2_O_2_ | 14.6 ± 1.6 | 15.6 ± 2.2 | 12.5 ± 2.1 | **7.9 ± 2.1 (*p* =0.002)** | 12.8 ± 2.2 | **21.6 ± 2.1 (*p* =0.001)** | **10.4 ± 2.1 (*p* = 0.046)** | 12.8 ± 2.1 | 14.4 ± 2.2 | **9.6 ±2.2 (*p* = 0.021)** | 14.5 ± 2.2 | 13.1 ± 2.1 |
| Lateral root count 0.5 mM H_2_O_2_ | 11.9 ± 1.2 | 13.2 ± 1.7 | 8.9 ± 1.7 | 9.8 ± 1.7 | 11.4 ± 1.7 | 9.9 ± 1.9 | 10.1 ± 1.9 | 10.1 ± 1.7 | 12.8 ± 1.7 | 12.0 ± 1.7 | 8.6 ± 2.0 | 10.4 ± 1.7 |
| Secondary root count 0 mM H_2_O_2_ | 12.4 ± 1.6 | 11.7 ± 1.6 | 13.5 ± 1.6 | 13.9 ± 1.7 | 11.3 ± 1.7 | 13.0 ± 1.7 | 11.3 ± 1.6 | 13.2 ± 1.6 | 13.0 ± 1.8 | 11.9 ± 1.7 | **8.9 ± 1.6 (*p* =0.036)** | **8.1 ± 1.6 (*p* = 0.009)** |
| Secondary root count 0.25 mM H_2_O_2_ | 12.6 ± 1.1 | 13.3 ± 1.6 | **8.3 ± 1.5 (*p* = 0.014)** | **7.2 ± 1.5 (*p* = 0.002)** | 10.6 ± 1.6 | **16.5 ± 1.5 (*p* = 0.005)** | **7.5 ± 1.5 (*p* = 0.002)** | 11.8 ± 1.5 | 11.6 ± 1.6 | 9.4 ± 1.6 | 12.2 ± 1.6 | 10.2 ± 1.5 |
| Secondary root count 0.5 mM H_2_O_2_ | 9.6 ± 0.8 | 10.8 ± 1.2 | **6.4 ± 1.2 (*p* = 0.007)** | 8.9 ± 1.2 | 9.4 ± 1.1 | 9.2 ± 1.3 | 8.2 ± 1.2 | 9.1 ± 1.1 | 9.7 ± 1.2 | 8.9 ± 1.1 | 8.0 ± 1.4 | 8.6 ± 1.1 |
| Tertiary root count 0 mM H_2_O_2_ | 2.2 ± 0.9 | 2.1 ± 1.3 | **5.6 ± 1.3 (*p* =0.008)** | 2.4 ± 1.3 | 2.0 ± 1.3 | 1.6 ± 1.3 | 4.6 ± 1.3 | 0.6 ± 1.3 | 1.2 ± 1.4 | 0.9 ± 1.3 | 1.5 ± 1.3 | 3.4 ± 1.3 |
| Tertiary root count 0.25 mM H_2_O_2_ | 2.5 ± 0.8 | 2.2 ± 1.1 | 4.2 ± 1.1 | 0.6 ± 1.1 | 2.2 ± 1.1 | **5.0 ± 1.1 (*p* = 0.028)** | 2.9 ± 1.1 | 1.1 ± 1.1 | 2.5 ± 1.1 | 0.3 ± 1.1 | 2.3 ± 1.1 | 2.9 ± 1.1 |
| Tertiary root count 0.5 mM H_2_O_2_ | 2.3 ± 0.6 | 3.0 ± 0.9 | 2.3 ± 0.9 | 0.9 ± 0.9 | 2.0 ± 0.9 | 0.7 ± 1.0 | 1.9 ± 1.0 | 1.1 ± 0.9 | 2.9 ± 0.9 | 3.0 ± 0.9 | 0.6 ± 1.1 | 1.7 ± 0.9 |

Table S3: Primers used to genotype the stock lines obtained from NASC.

| **Line** | **LP** | **RP** |
| --- | --- | --- |
| PIP1;1 | CTGCAAATTCCACCAAGATTG | TTGTAATCTTGGCACAAACCAG |
| PIP1;2 | TTCCATCATCTTCCAACCTTG | GAAGATGATTGCAGCTCCAAG |
| PIP1;3 | TAACGTGGCCCATAAAGAGTG | AATTGGTCTTTTGTTGCATGC |
| PIP2;2 | CAACCATAAGCCTACCAAAAGG | TTATAGATTACGGCAGCTCCG |
| PIP2;3 | TCGCTAAATTGTGTTTGCCTC | GAAACCGGTCCGGTTTATTAC |
| PIP2;4 | GAGCGGTCATAGCTGAGTTTG | ACTGATGGTAAAACGCTGCTG |
| PIP2;5 | GCCCATGATTGTAATGAATGG | ATGTACCAATGATCTCGGCTG |
| PIP2;6 | ACCATATTTGTGCTCCACGTC | AGTGATTGATAGGATGCACCG |

Table S4: Biomass and water content, means with SE, in bold significant differences compared to WT

|  | WT | *pip1;1* | *pip1;2* | *pip1;3* | *pip2;2* | *pip2;3* | *pip2;4* | *pip2;5* | *pip2;6* | *pip2;2x2;4* | *pip2;4x2;5* | *pip2;2x2;4x2;5* |
| --- | --- | --- | --- | --- | --- | --- | --- | --- | --- | --- | --- | --- |
| Root DW (g) control | 0.029 ± 0.004 | 0.023 ± 0.006 | 0.023 ± 0.006 | 0.024 ± 0.006 | 0.032 ± 0.006 | 0.019 ± 0.006 | 0.019 ± 0.006 | 0.033 ± 0.006 | 0.031 ± 0.006 | 0.027 ± 0.006 | 0.025 ± 0.006 | 0.021 ± 0.006 |
| Root DW (g) H_2_O_2_ | 0.012 ± 0.005 | 0.02 ± 0.006 | 0.022 ± 0.006 | 0.024 ± 0.006 | 0.024 ± 0.006 | 0.04 ± 0.006 | **0.014 ± 0.006 (*p* < 0.001)** | **0.025 ± 0.006 (*p* = 0.045)** | 0.012 ± 0.007 | **0.044 ± 0.006 (*p* < 0.001)** | **0.031 ± 0.007 (*p* = 0.006)** | 0.015 ± 0.006 |
| Shoot DW (g) control | 0.061 ± 0.005 | 0.058 ± 0.008 | 0.059 ± 0.008 | 0.067 ± 0.008 | 0.067 ± 0.008 | 0.059 ± 0.008 | 0.048 ± 0.008 | 0.062 ± 0.008 | 0.072 ± 0.008 | 0.075 ± 0.008 | **0.033 ± 0.008 (*p* < 0.001)** | 0.057 ± 0.008 |
| Shoot DW (g) H_2_O_2_ | 0.033 ± 0.005 | 0.037 ± 0.007 | **0.053 ± 0.007 (*p* = 0.007)** | 0.040 ± 0.007 | 0.038 ± 0.007 | **0.063 ± 0.007 (*p* < 0.001)** | 0.040 ± 0.007 | **0.052 ± 0.007 (*p* = 0.009)** | 0.036 ± 0.007 | **0.056 ± 0.007 (*p* = 0.001)** | **0.050 ± 0.008 (*p* = 0.017)** | 0.034 ± 0.007 |
| Total DW (g) control | 0.090 ± 0.008 | 0.083 ± 0.012 | 0.083 ± 0.012 | 0.093 ± 0.012 | 0.096 ± 0.012 | 0.078 ± 0.012 | **0.066 ± 0.012 (*p* = 0.045)** | 0.094 ± 0.012 | 0.104 ± 0.012 | 0.101 ± 0.012 | **0.058 ± 0.013 (*p* = 0.011)** | 0.079 ± 0.012 |
| Total DW (g) H_2_O_2_ | 0.045 ± 0.009 | 0.057 ± 0.012 | **0.075 ± 0.012 (*p* = 0.016)** | 0.063 ± 0.012 | 0.060 ± 0.012 | **0.112 ± 0.012 (*p* < 0.001)** | 0.054 ± 0.012 | **0.076 ± 0.012 (*p* = 0.013)** | 0.050 ± 0.013 | **0.097 ± 0.012 (*p* < 0.001)** | **0.081 ± 0.013 (*p* = 0.006)** | 0.048 ± 0.012 |
| R-S ratio control | 0.450 ± 0.082 | 0.408 ± 0.116 | 0.394 ± 0.119 | 0.372 ± 0.116 | 0.481 ± 0.116 | 0.315 ± 0.116 | 0.391 ± 0.116 | 0.533 ± 0.116 | 0.399 ± 0.116 | 0.378 ± 0.116 | **0.914 ± 0.123 (*p* < 0.001)** | 0.355 ± 0.116 |
| R-S ratio H_2_O_2_ | 0.361 ± 0.081 | 0.527 ± 0.114 | 0.465 ± 0.114 | 0.470 ± 0.114 | **0.687 ± 0.114 (*p* = 0.005)** | **0.662 ± 0.114 (*p* = 0.010)** | 0.329 ± 0.114 | 0.472 ± 0.114 | 0.369 ± 0.114 | **0.784 ± 0.114 (*p* < 0.001)** | 0.573 ± 0.121 | 0.397 ± 0.114 |
| Root RWC control | 63.1 ± 4.1 | **82.3 ± 5.7 (*p* = 0.001)** | **77.2 ± 5.9 (*p* = 0.018)** | **87.5 ± 5.9 (*p* < 0.001)** | 70.1 ± 5.7 | **86.4 ± 5.7 (*p* < 0.001)** | **89.9 ± 6.1 (*p* < 0.001)** | **77.4 ± 5.7 (*p* = 0.014)** | 66.1 ± 6.3 | **82.5 ± 5.7 (*p* < 0.001)** | 59.8 ± 6.3 | **81.7 ± 5.7 (*p* = 0.002)** |
| Root RWC H_2_O_2_ | 86.7 ± 4.2 | **74.0 ± 6.1 (*p* = 0.042)** | 86.1 ± 6.0 | 90.4 ± 6.4 | **72.4 ± 5.8 (*p* = 0.016)** | 74.6 ± 6.1 | 85.4 ± 6.0 | 79.0 ± 6.1 | 88.6 ± 6.0 | **52.8 ± 5.8 (*p* < 0.001)** | 77.0 ± 6.7 | 76.8 ± 6.0 |
| Shoot RWC control | 85.9 ± 0.8 | **90.1 ± 1.1 (*p* < 0.001)** | **90.3 ± 1.1 (*p* < 0.001)** | **89.7 ± 1.1 (*p* < 0.001)** | **88.7 ± 1.1 (*p* = 0.012)** | **91.0 ± 1.1 (*p* < 0.001)** | **91.0 ± 1.1 (*p* < 0.001)** | **88.1 ± 1.1 (*p* = 0.050)** | 87.8 ± 1.2 | 86.0 ± 1.1 | **89.8 ± 1.2 (*p* < 0.001)** | 87.8 ± 1.1 |
| Shoot RWC H_2_O_2_ | 89.4 ± 1.5 | 87.6 ± 2.0 | 89.2 ± 2.1 | 88.8 ± 2.1 | 89.5 ± 2.1 | **84.2 ± 2.0 (*p* = 0.012)** | 89.3 ± 2.1 | 86.6 ± 2.1 | 88.6 ± 2.0 | **80.3 ± 2.1 (*p* < 0.001)** | **81.4 ± 2.1 (*p* < 0.001)** | 85.8 ± 2.1 |
| Whole plant RWC control | 82.7 ± 1.3 | **88.7 ± 1.9 (*p* = 0.002)** | **88.4 ± 1.9 (*p* = 0.003)** | **88.8 ± 1.9 (*p* = 0.001)** | 85.8 ± 1.9 | **90.3 ± 1.9 (*p* < 0.001)** | **89.9 ± 1.9 (*p* < 0.001)** | 86.5 ± 1.9 | 82.4 ± 1.9 | 85.3 ± 1.9 | 85.6 ± 2.1 | **86.8 ± 1.9 (*p* = 0.029)** |
| Whole plant RWC H_2_O_2_ | 88.9 ± 2.0 | 85.0 ± 2.8 | 87.7 ± 2.9 | 89.6 ± 3.0 | **83.3 ± 2.8 (*p* = 0.050)** | 83.6 ± 3.0 | 88.8 ± 2.9 | 84.3 ± 2.9 | 88.7 ± 2.9 | **72.1 ± 2.9 (*p* < 0.001)** | **78.7 ± 3.0 (*p* < 0.001)** | 84.1 ± 2.9 |
| AWC leaf control | 0.43 ± 0.05 | 0.51 ± 0.07 | 0.55 ± 0.07 | **0.59 ± 0.07** | 0.51 ± 0.07 | **0.60 ± 0.07** | 0.54 ± 0.07 | 0.45 ± 0.07 | 0.45 ± 0.07 | 0.43 ± 0.07 | 0.30 ± 0.07 | 0.40 ± 0.07 |
| AWC leaf H_2_O_2_ | 0.25 ± 0.03 | 0.29 ± 0.04 | **0.37 ± 0.04** | 0.27 ± 0.04 | 0.25 ± 0.04 | **0.34 ± 0.04** | 0.30 ± 0.04 | 0.32 ± 0.04 | 0.26 ± 0.04 | 0.22 ± 0.04 | 0.22 ± 0.04 | 0.20 ± 0.04 |

Table S5: Primers for PIP and references genes used in the qRT-PCR.

| **PIP Gene** | **Primer** |
| --- | --- |
| PIP1;1 (At3g61430) | For: 5´- TCTTAACCCAAAGGCCAACA-3´ |
| PIP1;1 (At3g61430) | Rev: 5´- CAGAATCCAGCACAATACCG-3´ |
| PIP1;2 (At2g45960) | For: 5´- TGGGATGACCACTGGGTGTTT-3´ |
| PIP1;2 (At2g45960) | Rev: 5´- GGGATGGCTCTGATGACAACC-3´ |
| PIP1;3 (At1g01620) | For: 5´- CTTCCGTCGGAATCCAAGGT-3´ |
| PIP1;3 (At1g01620) | Rev: 5´- CGTGTGAGCGAAAGCTTCCT-3´ |
| PIP1;4 (At4g00430) | For: 5´- GGAATCTCTGGTGGGCACAT-3´ |
| PIP1;4 (At4g00430) | Rev: 5´- CTCCGAGACATTGCATCACG-3´ |
| PIP1;5 (At4g23400) | For: 5´- TGGGATGACCATTGGATTTT-3´ |
| PIP1;5 (At4g23400) | Rev: 5´- TCTGGACCGTGGAATCTTTC-3´ |
| PIP2;1 (At3g53420) | For: 5´- GCTGGAATCTCAGGAGGACATATT-3´ |
| PIP2;1 (At3g53420) | Rev: 5´- AGCTCCAAGGCACTGCATTACT-3´ |
| PIP2;2 (At2g37170) | For: 5´- TAGATTGCGGCGGAGTTGGA-3´ |
| PIP2;2 (At2g37170) | Rev: 5´- CGCTGGGTTAATGTGACCACCA-3´ |
| PIP2;3 (At2g37180) | For: 5´- CAAAGACGTGGAAGGACCTGAG-3´ |
| PIP2;3 (At2g37180) | Rev: 5´- ACTTGGTAAGCTCGTCCGCA-3´ |
| PIP2;4 (At5g60660) | For: 5´- TTCGACGCAGAGGAGCTTAC-3´ |
| PIP2;4 (At5g60660) | Rev: 5´- GCTACGAACTCGGCGATGACT-3´ |
| PIP2;5 (At3g54820) | For: 5´- CCGATGGCTACAACAAAGGT-3´ |
| PIP2;5 (At3g54820) | Rev: 5´- CACGTGAGAGTCACGAGCAT-3´ |
| PIP2;6 (At2g39010) | For: 5´- TGGTGGGCATATTAATCCGGCAGT-3´ |
| PIP2;6 (At2g39010) | Rev: 5´- TGACCAAAGCCACACCACAAATGG-3´ |
| PIP2;7 (At4g35100) | For: 5´- GGCATCTCTGGTGGACACATC-3´ |
| PIP2;7 (At4g35100) | Rev: 5´- CAACTCCACAAGTGGCTCCG-3´ |
| PIP2;8 (At2g16850) | For: 5´- CGTGGGATGACCAATGGATC-3´ |
| PIP2;8 (At2g16850) | Rev: 5´- TGCGTTGCTTCGGAACGAG-3´ |
| **Reference Gene** | |
| YLS8 (At5g08290) | For: 5´ - TTACTGTTTCGGTTGTTCTCCATTT-3´ |
| YLS8 (At5g08290) | Rev: 5´ - CACTGAATCATGTTCGAAGCAAGT-3´ |
| MON1 (At2g28390) | For: 5´ - CAGACAAGGCGATGGCGATA-3´ |
| MON1 (At2g28390) | Rev: 5´ - GCTTTCTCTCAAGGGTTTCTGGGT-3´ |
| TIP41 (At4g34270) | For: 5´ - GTGAAAACTGTTGGAGAGAAGCAA-3´ |
| TIP41 (At4g34270) | Rev: 5´ - TCAACTGGATACCCTTTCGCA-3´ |
